# Inverted Meckel’s Diverticulum: Rare Etiology of an Intestinal Obstruction

**DOI:** 10.7759/cureus.1806

**Published:** 2017-10-27

**Authors:** Vishnu R Mani, Aleksandr Kalabin, Anant Dinesh, Ajai Rajabalan, Marina Landa, Albert Adu

**Affiliations:** 1 Department of Surgery, New York University School of Medicine, and the Laura and Isaac Perlmutter Cancer Center, Columbia University School of Physicians and Surgeons at Harlem Hospital Center; 2 Department of General Surgery, Columbia University College of Physicians and Surgeons at Harlem Hospital Center; 3 Department of Medicine, North Side Medical Center; 4 Pathology, Columbia University College of Physicians and Surgeons at Harlem Hospital Center

**Keywords:** intestinal obstruction, meckels diverticulum, ectopic, gastric, pancreatic

## Abstract

Acute gastrointestinal obstruction can have a varied spectrum of clinical presentation and etiologies. It has been studied in detail and the management criteria have been well defined for the most part in our era. The etiologies are usually well defined. However, acute small bowel obstruction (SBO) due to intussusception caused by an inverted Meckel's diverticulum is a rare phenomenon that is often times missed on initial presentation and/or consequently until resected and visualized on pathological examination. We present a case of a 34-year-old presenting with symptoms and signs of acute intestinal obstruction and radiographic exam showing ileo-ileal intussusception. The patient failed to improve initially following conservative management and was taken to the operating room for small bowel resection which then revealed an inverted Meckel’s diverticulum.

## Introduction

Small bowel obstruction is more common than colon obstruction, and its incidence is about 350,000 per year in the US. It comprises about 15% of all acute surgical gastrointestinal admissions and about the same proportion of emergency admissions for abdominal pain [1]. Over the years, we have delineated the most common etiology of SBOs as adhesions, incarcerated hernia, malignancy, and rarely volvulus and etiologies leading to intussusception such as polyps, Meckel’s diverticulum, and other diverticula which may serve as a lead point. Inverted Meckel’s diverticulum is a rare pathology, and it being a cause for small bowel obstruction is rarer. We hereby report a case of a small bowel obstruction due to intussusception which on pathology was found to be inverted Meckel’s diverticulum.

## Case presentation

**Figure 1:**
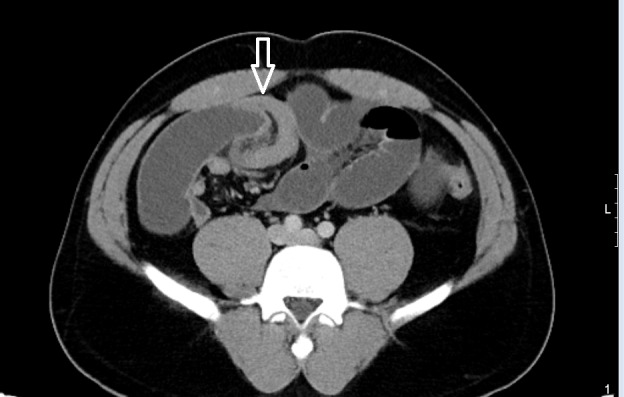
Computed tomography of abdomen - coronal plane

**Figure 2:**
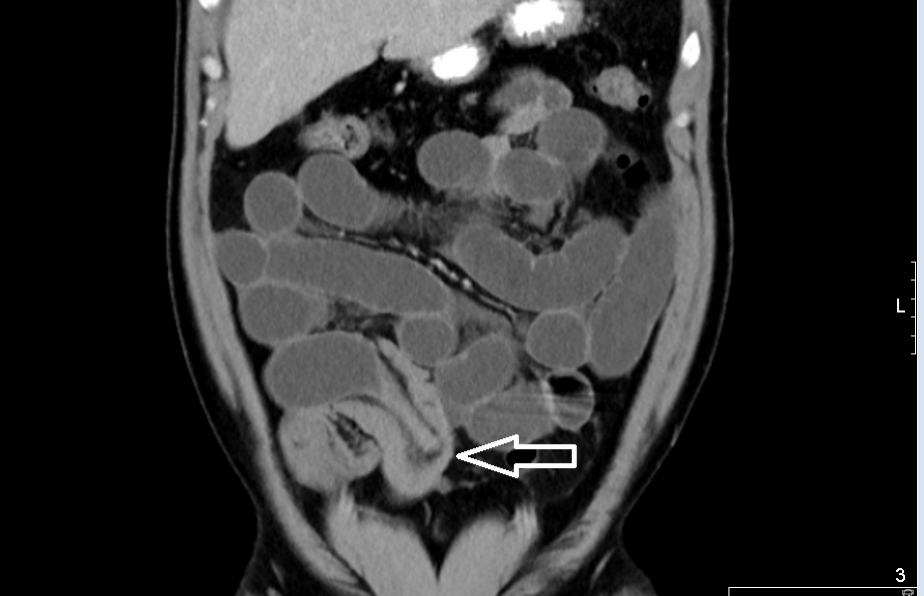
Computed tomography of abdomen - sagittal plane

A 34-year-old male with a past medical history of gastroesophageal reflux disorder presented to the emergency room with diffuse crampy abdominal pain since four months progressively worsening over two to three days, which was associated with nausea, bilious vomiting, and obstipation. Physical examination was notable for abdominal distention and tenderness in the right lower quadrant with no significant guarding or rigidity. A nasogastric tube was placed for decompression and the patient was resuscitated with intravenous fluids with moderate improvement of symptoms. Laboratory tests were unremarkable. Computed tomography (CT) of the abdomen and pelvis was performed, which showed right lower quadrant ileo-ileal intussusception with hypodensity distal to the collapsed distal ileum (Figures 1-2).

The patient was scheduled for emergent operative intervention. Exploratory laparotomy was performed and bowel was inspected. Small bowel loops were found dilated till the distal ileum where an intussusception was identified approximately 40 cm proximal to the ileocecal valve with a discrete palpable mass within the ileum. En bloc resection of the small bowel with adequate margin was performed and side-to-side (functional end-to-end) anastomosis was done using a stapling device. The rest of the bowel was healthy with no evidence of necrosis or ischemia. Postoperative hospital course was uneventful and he was advanced to a regular diet on the third postoperative day and discharged home on the fourth postoperative day.

**Figure 3:**
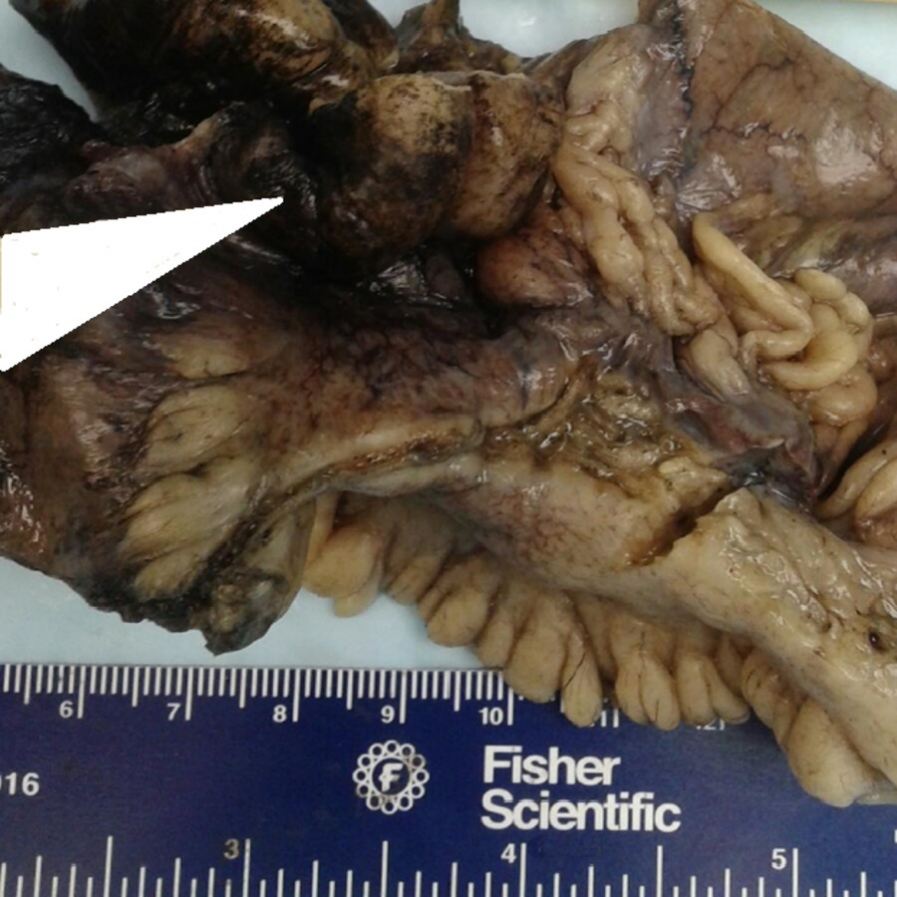
Gross picture depicting the serosal surface

Gross pathology of the specimen revealed a firm, extraverted, elongated invagination into the ileum measuring 5.5 cm in its greatest dimension, covered with a smooth mucosal lining on the luminal surface with an irregular fatty tail of tissue on the serosal surface (Figure 3).

**Figure 4:**
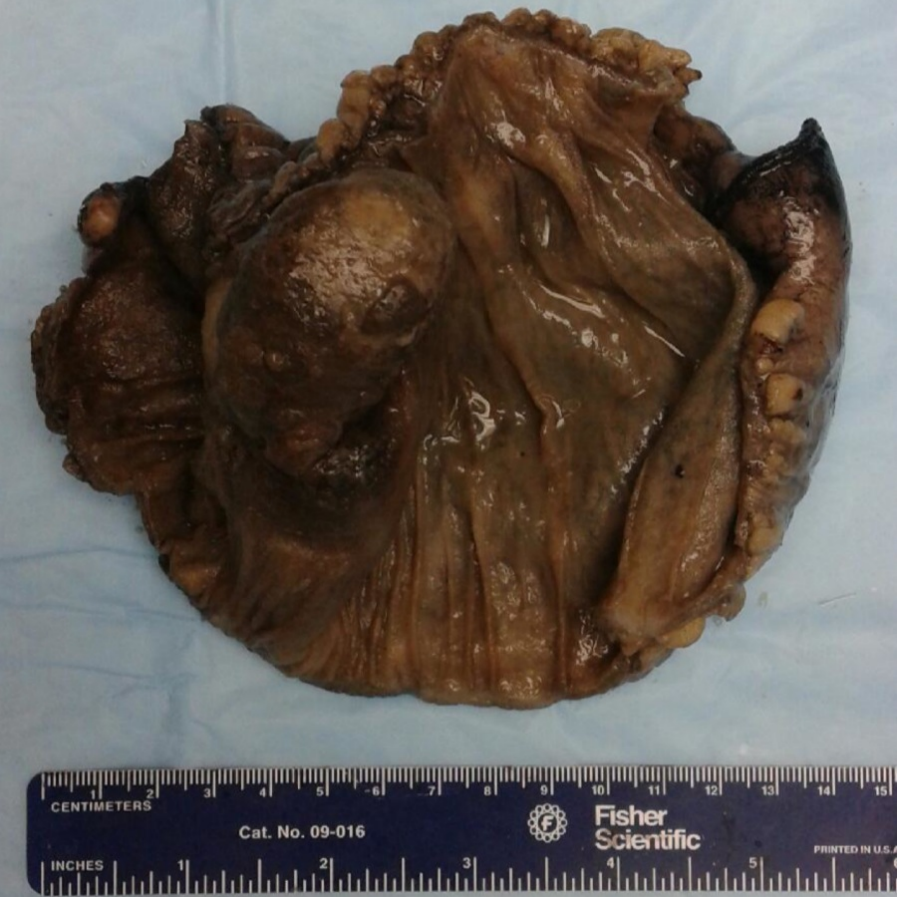
Intraluminal picture depicting the inverted diverticulum

 The cut surface of the mass was firm and yellow in color (Figure 4).

**Figure 5:**
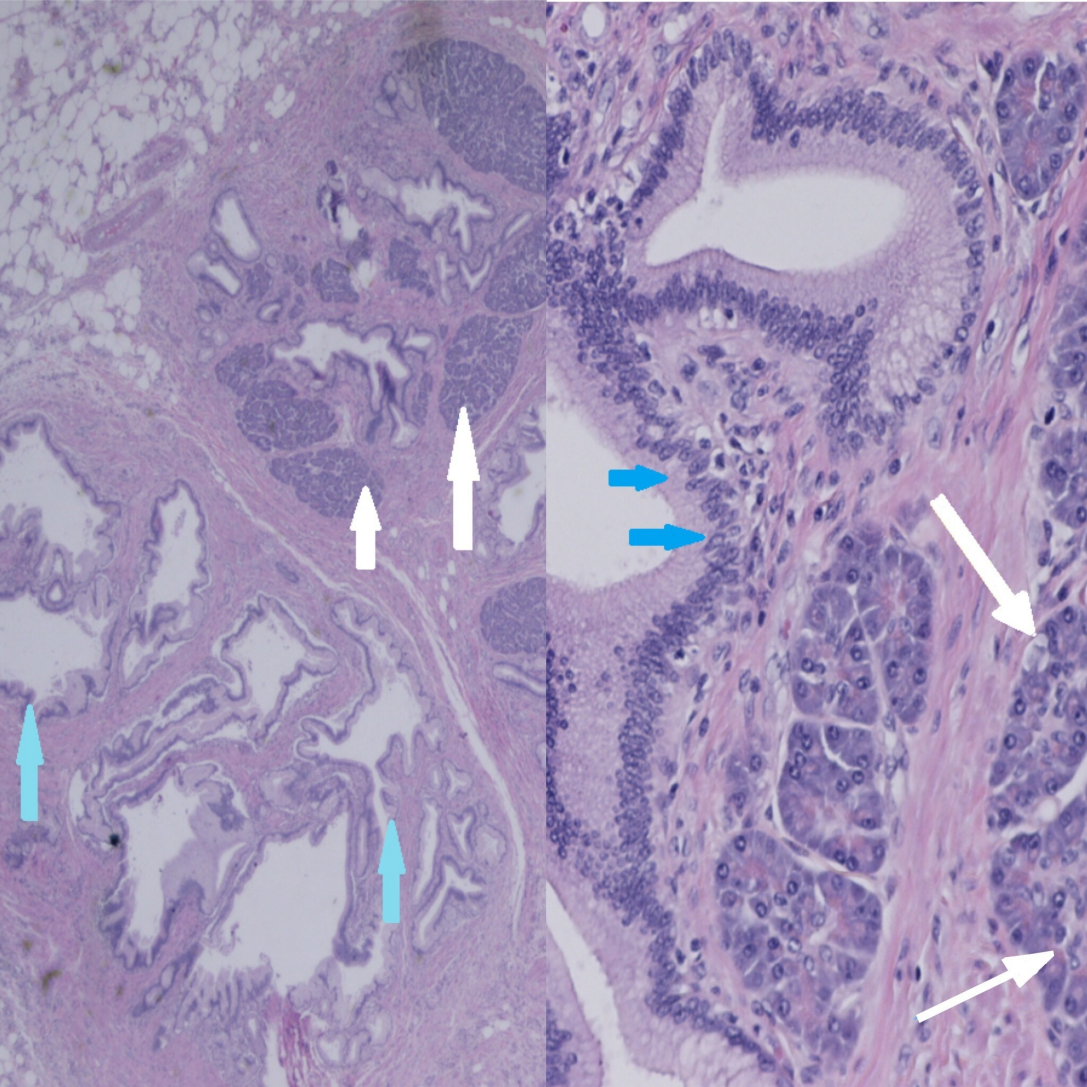
Histology of the specimen clearly indicating the presence of pancreatic and gastric tissue

Microscopic examination showed benign fat and incorporated heterotopic pancreatic and gastric tissue with no atypical or dysplastic features. Follow-up course was unremarkable (Figure 5).

## Discussion

Small bowel obstruction is one of the most common reasons for acute abdomen emergencies and is likely to involve a surgical admission. Bowel obstructions are commonly caused by postoperative adhesions, neoplasms, hernias, volvulus, intussusception, or inflammatory bowel diseases. If not treated promptly, bowel obstruction can lead to acute intestinal dilatation and perforation with consequent faecal peritonitis, sepsis, and multiple organ failure, which have high morbidity and mortality. One of the uncommon causes of bowel obstruction is intussusception (telescoping), when a loop of the small bowel with its mesentery invaginates into the lumen of the bowel segment distal to it. Although it is a common abdominal emergency in children younger than two years of age, it is unusual in adults and the diagnosis is commonly overlooked. Adult intussusception represents 1% of all bowel obstructions, and 0.003%-0.02% of all hospital admissions [2]. Adult intussusception is a completely different entity than in children, as most pediatric cases are considered to be idiopathic with no clear pathological lead point; however, a pathologic etiology is identified in a majority of adult cases. Benign or malignant tumors serving as a lead point are the most common causes of small bowel intussusception in adults presenting with obstructive symptoms. In postoperative patients, intussusceptions may be related to suture lines, adhesions, or intestinal tubes [3]. Statistically benign or malignant neoplasms explain two-thirds of cases of intussusception (50%-75%) with a lead point, and the remaining cases are caused by infections, postoperative adhesions, Crohn granulomas, intestinal ulcers (Yersinia), and very rarely congenital abnormalities such as Meckel's diverticulum [4]. In one recently published retrospective study, all patients admitted from 2006 to 2014 with a diagnosis of intestinal intussusception were reviewed. Eight patients presented with signs and symptoms of acute intestinal obstruction that required hospitalization and subsequent surgery. Diagnosis of intussusception was confirmed intraoperatively in all patients, and pathological examination identified two cases of ileal lipoma (25%) and cecal lipoma (25%) each, and one case each of an adenomatous polyp of the ileum (12.5%), cecal adenocarcinoma (12.5%), ileal teratoma (12.5%), and jejunal stromal tumor (12.5%) [5]. In another retrospective study comprising 107 patients diagnosed with intussusception reviewed over a ten-year period, 30 patients had their condition managed conservatively, and the rest of the 77 patients underwent surgery. Pathological causes of intussusception were identified in 62 patients (80.5%). Benign pathologies were seen in 41 patients (53.2%), malignant pathologies in 21 patients (27.2%), and unknown pathologies in 15 patients (19.4%). The patients with unknown pathologies included 11 with “idiopathic” intussusception of which four patients underwent manual reduction [6].

Neither study clearly showed the true incidence of Meckel’s diverticulum as a cause of adult intussusception. Ectopic tissue such as gastric or pancreatic mucosa is found in about 50%-57% of patients with Meckel’s diverticulum. This could be explained by embryologic origin and the development of midgut. Rutherford et al. studied 147 surgical specimens and found heterotopic tissue in 57%. The types of ectopic tissue found were gastric, pancreatic, colonic, jejunal, and duodenal [7]. The most common complication of Meckel’s diverticulum is hemorrhage, which is reported in about 50% of patients with symptoms associated with the diverticulum, especially in the pediatric population.

Usually, the mechanism of bleeding is the ulceration of the adjacent anti-mesenteric wall of the small bowel due to acid secretion by the ectopic gastric mucosa within the diverticulum, but not from the mucosa or ectopic tissue within it. Intestinal obstruction is another common complication most commonly seen in adults and may be caused by volvulus of the small bowel around a diverticulum, intussusception, or incarceration of the diverticulum in a hernia (Littre′s hernia) and enterolith formation in the diverticulum. Meckel’s diverticulitis most commonly presented as acute appendicitis usually accounts for 10%-20% of complications and is more common in older patients. Benign as well as malignant diverticulum neoplasm also have been reported as a complication of Meckel’s diverticulum. Common benign neoplasms include lipoma, leiomyoma, neurofibroma, and angioma, while malignant tumors include leiomyosarcoma and carcinoid, which represent about 80% of such lesions while adenocarcinoma and metastatic lesions constitute the remainder. Identified over a 15-year period, only 15 patients presented with complicated Meckel’s diverticulum. Inflammation (20.0%), obstructive ileus (20%), intussusception (13.3%), perforation (26.7%) and strangulated Littre’s hernia were most common complications [8].

Although Meckel’s diverticulum is the most common congenital anomaly of the small bowel, inversion or invagination of the diverticulum is a rare occurrence and, if detected, warrants surgical management due to high risk of complications such as ileo-ileal intussusception. To date, there are only a few reported cases in the literature that clearly demonstrate inverted Meckel’s diverticulum as a lead point in bowel ‘telescoping’ and subsequent bowel obstruction. Inverted Meckel’s diverticulum also presents clinically as lower gastrointestinal bleeding, chronic abdominal pain, or with symptoms and signs suggestive of small bowel obstruction. The most common age group of presentation is among young adults unlike Meckel’s diverticulum, and generally present as subacute or chronic symptoms including abdominal pain or low-grade small bowel obstruction [9].

Initial workup in patients with clinical signs of obstruction includes abdominal X-ray, which may suggest nonspecific bowel pattern. Barium studies have been reported to diagnose this pathology in around two-thirds of patients with inverted Meckel’s diverticulum. Specific findings seen on barium examination include elongated smoothly marginated intraluminal mass parallel to the long axis of the bowel. Inverted bulbous tip produces a characteristic club-like appearance seen in more than 90% cases. This finding is due to mesenteric fat filling at the tip of the inverted sac. CT findings are characteristic for central area of fat attenuation surrounded by a thick collar of soft tissue attenuation [10].

## Conclusions

Meckel’s diverticulum can invert into the intestinal lumen, and there are reported cases; however, the exact mechanism is not clearly understood. We hypothesized that persistent ectopic tissue may cause abnormal peristaltic movement that subsequently might invert the diverticulum. Once inverted, it can serve as a lead point for further telescoping culminating in an intussusception, intestinal obstruction, segmental ischemia, or even perforation. Preoperative diagnosis of inverted Meckel's diverticulum may be challenging due to overlapping clinical and imaging features of other surgical conditions of the abdomen. And clinicians should keep this rare entity in mind while evaluating patients presenting with signs and symptoms of intussusception to avoid excess morbidity.

## References

[1] Rami Reddy SR, Cappell MS (2017). A systematic review of the clinical presentation, diagnosis, and treatment of small bowel obstruction. Curr Gastroenterol Rep.

[2] Azar T, Berger DL (1997). Adult intussusception. Ann Surg.

[3] Gore RM, Silvers R, Thakrar KH (2015). Bowel obstruction. Radiol Clin.

[4] Marsicovetere P, Ivatury J, White B, Holubar SD (2017). Intestinal intussusception: etiology, diagnosis, and treatment. Clin Colon Rectal Surg.

[5] Maghrebi H, Makni A, Rhaiem R (2017). Adult intussusceptions: clinical presentation, diagnosis and therapeutic management. Int J Surg Case Rep.

[6] Kim JW, Lee BH, Park SG, Kim BC, Lee S, Lee SJ (2017). Factors predicting malignancy in adult intussusception: an experience in university-affiliated hospitals. Asian J Surg.

[7] Rutherford RB, Akers DR (1966). Meckel's diverticulum: a review of 148 pediatric patients, with special reference to the pattern of bleeding and to mesodiverticular vascular bands. Surgery.

[8] Matsagas MI, Fatouros M, Koulouras B, Giannoukas AD (2005). Incidence, complications, and management of Meckel's diverticulum. Arch Surg.

[9] Pantongrag-Brown L, Levine MS, Buetow PC, Buck JL, Elsayed AM (1996). Meckel's enteroliths: clinical, radiologic, and pathologic findings. AJR Am J Roentgenol.

[10] Gaisie G, Kent C, Klein L, Schreiber H (1993). Radiographic characteristics of isolated invaginated Meckel's diverticulum. Pediatr Radiol.

